# Comparison of 2 chromogenic media for the detection of extended-spectrum β-lactamase producing Enterobacteriaceae stool carriage in nursing home residents

**DOI:** 10.1016/j.diagmicrobio.2015.11.008

**Published:** 2016-03

**Authors:** Beth Blane, Hayley J. Brodrick, Theodore Gouliouris, Kirsty E. Ambridge, Angela D. Kidney, Catherine M. Ludden, Direk Limmathurotsakul, M. Estée Török, Sharon J. Peacock

**Affiliations:** a Department of Medicine, University of Cambridge, Cambridge, United Kingdom; b Public Health England, Clinical Microbiology and Public Health Laboratory, Cambridge, United Kingdom; c Cambridge University Hospitals NHS Foundation Trust, Cambridge, United Kingdom; d Mahidol-Oxford Tropical Medicine Research Unit, Mahidol University, Bangkok, Thailand

**Keywords:** Extended-spectrum β-lactamase, Enterobacteriaceae, Detection, Chromogenic agar, Sensitivity, Selectivity

## Abstract

ChromID ESBL agar and *Brilliance* ESBL agar were compared for the isolation of extended-spectrum β-lactamase (ESBL)–producing Enterobacteriaceae from 298 stools. These had comparable sensitivity and selectivity for the 116 positive samples. Pre-enrichment with cefpodoxime and extending incubation to 48 hours after direct plating both significantly increased sensitivity but reduced selectivity of both agars.

## Highlights

• Two chromogenic agars were compared for the isolation of ESBL Enterobacteriaceae.
• Both agars had comparable sensitivity and selectivity.
• Sensitivity after direct plating was higher when incubated for 48 versus 24 hours.
• Using pre-enrichment with 24-hour incubation further increased the sensitivity.
• There was a loss of selectivity after 48-hour incubation for all methods

The incidence of bloodstream infections caused by extended-spectrum β-lactamase (ESBL)–producing *Escherichia coli* is rising in Europe and the United States (Castanheira et al., 2010, European Centre for Disease Prevention and Control, 2014). This trend is mirrored by the global dissemination of other ESBL-producing species, particularly *Klebsiella* spp. (WHO, 2014). Infections caused by ESBL-producing Enterobacteriaceae are associated with increased mortality, length of stay, and hospital costs compared to non–ESBL-producing Enterobacteriaceae (Schwaber and Carmeli, 2007, Tumbarello et al., 2010). Screening for rectal carriage of ESBL-producing organisms has been advocated as an infection control strategy particularly during outbreaks (Siegel et al., 2006) and is routinely performed in some healthcare settings (Murk et al., 2009). The advent of chromogenic media has improved the detection of ESBL-producing Enterobacteriaceae (Glupczynski et al., 2007, Huang et al., 2010, Réglier-Poupet et al., 2008). However, few studies have assessed the yield of target organisms using different culture methodologies with conflicting results reported on the value of prolonging direct incubation to 48 hours (Réglier-Poupet et al., 2008, Willems et al., 2013) or of using a pre-enrichment step (Diederen et al., 2012, Murk et al., 2009). The aim of this method comparison study was to assess the sensitivity and selectivity of 2 chromogenic media with or without pre-enrichment to detect ESBL-producing Enterobacteriaceae in stool during a longitudinal carriage survey of ESBL-producing organisms in residents of a nursing home in Cambridgeshire. The study protocol was approved by the National Research Ethics Service East of England Ethics Committee (NRES ref: 13/LO/1278) and the Cambridge University Hospitals NHS Foundation Trust Research and Development Department. Written informed consent was obtained from participants or consultees, as appropriate.

A total of 298 stool samples were collected from 37 residents over a 14-week period, commencing in March 2014. Stools were refrigerated and processed within 24 hours, unless collected over the weekend when they were processed within 72 hours. We performed direct plating onto chromID ESBL agar (bioMérieux, Marcy l’Etoile, France) and *Brilliance* ESBL agar (Oxoid, Basingstoke, UK). In addition, we plated onto both media after a selective pre-enrichment step using cefpodoxime (Health Protection Agency, 2012). For direct plating, approximately 0.2 g of stool was plated onto each agar. For selective pre-enrichment, approximately 0.2 g of stool was added to 10 mL of Tryptic Soy Broth (Sigma, Dorset, UK) containing cefpodoxime 1 μg/mL (Oxoid), vortexed, and incubated with shaking at 150 rpm at 37 °C in air overnight. The following day, 200 μL of suspension was inoculated onto each agar. All agar plates were incubated at 37 °C in air and inspected after 24 and 48 hours. Presumptive Enterobacteriaceae were selected from chromID and *Brilliance* ESBL agars based on colony colour as defined by the manufacturers' instructions. Subcultured isolates were initially identified using matrix-assisted laser desorption/ionisation time-of-flight mass spectrometry (MALDI-TOF MS, Biotyper version 3.1, SR_Clinical library) (Bruker Daltonics, Bremen, Germany) with a MALDI-TOF MS score of 2.0 or higher. Bacterial species and the presence of genes encoding ESBL were subsequently confirmed from whole genome sequence data. DNA was extracted using the QIAxtractor (Qiagen), according to the manufacturer's instructions. Library preparation was conducted according to the Illumina protocol, and sequencing was performed on an Illumina HiSeq2000 with 100-cycle paired-end runs. Sequence reads were mapped using SMALT (http://www.sanger.ac.uk/resources/software/smalt/) to relevant reference genomes (*Klebsiella pneumoniae* NTUH K2044 [GenBank accession number NC_006625.1], and *E. coli* EC958 [GenBank accession number NZ_HG941718]). Molecular confirmation of the presence of at least 1 gene known to encode ESBL was sought by in silico PCR using the ResFinder database (Zankari et al., 2012). Sequence data for all isolates have been submitted to the European Nucleotide Archive (www.ebi.ac.uk/ena) with the accession numbers shown in Supplementary Table 1. Antimicrobial susceptibility testing was performed using the N206 card on the Vitek 2 instrument (bioMérieux) calibrated against EUCAST breakpoints. ESBL production was confirmed using the AmpC and ESBL detection disc test D68C (Mast Group, Merseyside, UK) as per the manufacturer's instructions. Colonies with a colour/morphology combination that were not consistent with Enterobacteriaceae growing on either media were examined by Gram stain and microscopy with additional identification to species level using MALDI-TOF MS, as required.

A total of 298 stool samples were collected from 37 residents over a 14-week period, commencing in March 2014. Stools were refrigerated and processed within 24 hours, unless collected over the weekend when they were processed within 72 hours. We performed direct plating onto chromID ESBL agar (bioMérieux, Marcy l’Etoile, France) and *Brilliance* ESBL agar (Oxoid, Basingstoke, UK). In addition, we plated onto both media after a selective pre-enrichment step using cefpodoxime (Health Protection Agency, 2012). For direct plating, approximately 0.2 g of stool was plated onto each agar. For selective pre-enrichment, approximately 0.2 g of stool was added to 10 mL of Tryptic Soy Broth (Sigma, Dorset, UK) containing cefpodoxime 1 μg/mL (Oxoid), vortexed, and incubated with shaking at 150 rpm at 37 °C in air overnight. The following day, 200 μL of suspension was inoculated onto each agar. All agar plates were incubated at 37 °C in air and inspected after 24 and 48 hours. Presumptive Enterobacteriaceae were selected from chromID and *Brilliance* ESBL agars based on colony colour as defined by the manufacturers' instructions. Subcultured isolates were initially identified using matrix-assisted laser desorption/ionisation time-of-flight mass spectrometry (MALDI-TOF MS, Biotyper version 3.1, SR_Clinical library) (Bruker Daltonics, Bremen, Germany) with a MALDI-TOF MS score of 2.0 or higher. Bacterial species and the presence of genes encoding ESBL were subsequently confirmed from whole genome sequence data. DNA was extracted using the QIAxtractor (Qiagen, Hilden, Germany), according to the manufacturer's instructions. Library preparation was conducted according to the Illumina protocol, and sequencing was performed on an Illumina HiSeq2000 with 100-cycle paired-end runs. Sequence reads were mapped using SMALT (http://www.sanger.ac.uk/resources/software/smalt/) to relevant reference genomes (*Klebsiella pneumoniae* NTUH K2044 [GenBank accession number NC_006625.1], and *E. coli* EC958 [GenBank accession number NZ_HG941718]). Molecular confirmation of the presence of at least 1 gene known to encode ESBL was sought by in silico PCR using the ResFinder database (Zankari et al., 2012). Sequence data for all isolates have been submitted to the European Nucleotide Archive (www.ebi.ac.uk/ena) with the accession numbers shown in Supplementary Table 1. Antimicrobial susceptibility testing was performed using the N206 card on the Vitek 2 instrument (bioMérieux) calibrated against EUCAST breakpoints. ESBL production was confirmed using the AmpC and ESBL detection disc test D68C (Mast Group, Merseyside, UK) as per the manufacturer's instructions. Colonies with a colour/morphology combination that were not consistent with Enterobacteriaceae growing on either media were examined by Gram stain and microscopy with additional identification to species level using MALDI-TOF MS, as required.

Sensitivity of detection of ESBL-producing Enterobacteriaceae was calculated by comparing the number of positive plates for a given method with the cumulative yield of ESBL-producing organisms from all 4 methods (direct plating onto 2 media and plating onto both following a pre-enrichment step). Selectivity for each method was defined as the number of plates that suppressed the growth of organisms that were not ESBL producers. Statistical analysis was performed using STATA, version 12.1 (STATA, College Station, TX, USA). Sensitivity and selectivity were compared using the exact McNemar test.

ESBL-producing organisms were isolated from 116/298 (39%) stool samples. Fifteen of the 37 residents tested were positive on at least 1 occasion (range 2–15 positive samples, median 8). All 116 isolates were positive for the *bla*_CTX-M-15_ gene, and 2 isolates also carried the *bla*_SHV-28_ gene. The majority were *E*. *coli* (111/116, 96%), and the remainder were *K*. *pneumoniae* (5/116, 4%). No stool samples grew more than 1 ESBL-producing species. Sensitivity of detection following direct plating was higher when incubated for 48 hours versus 24 hours (75% versus 63% for chromID agar, *P* = 0.0001; 68% versus 59% for *Brilliance* agar, *P* = 0.001). Using pre-enrichment with 24-hour incubation further increased the sensitivity of detection of ESBL-producing organisms (from 75% to 97% for chromID agar, *P* < 0.0001; and from 68% to 98% for *Brilliance* agar, *P* < 0.0001). We speculate that pre-enrichment improved sensitivity due to carriage of low levels of ESBL-producing organisms, although bacterial load was not formally quantified. Increasing the duration of incubation to 48 hours after a pre-enrichment step did not increase the detection rate further (*P* > 0.99 for both agars). Although the sensitivity to detect ESBL producers using chromID agar was higher than *Brilliance* agar for the direct plating method with 48-hour incubation (75% versus 68%, *P* = 0.04), the sensitivity was comparable for the enrichment method either with 24-hour or 48-hour incubation (both *P* = 0.50) (Table 1).

Selectivity was lower at 48 hours versus 24 hours after direct plating for both media (from 70% versus 85% for chromID agar, *P* < 0.0001; 64% versus 87% for *Brilliance* agar, *P* < 0.0001). Selectivity further declined when a pre-enrichment step was added followed by plating and 24-hour incubation for chromID agar (70–63%, *P* = 0.02) and a similar trend was observed for *Brilliance* agar (64–61%, *P* = 0.26). Increasing the duration of incubation to 48 hours after a pre-enrichment step significantly decreased the selectivity for both agars (from 63% to 55% for chromID agar, *P* < 0.0001, and from 61% to 58% for *Brilliance* agar, *P* = 0.002). Although selectivity of chromID agar was higher than *Brilliance* agar for the direct plating method with 48-hour incubation (70% versus 64%, *P* = 0.02), their selectivity was comparable for the enrichment method either with 24-hour or 48-hour incubation (both *P* > 0.20).

Both media supported the growth of a range of species that were not ESBL-producing Enterobacteriaceae. The majority were *Pseudomonas* spp. (Table 2), although these were distinguishable based on colour and colonial morphology. The most problematic group was a small number of Enterobacteriaceae that displayed the expected phenotype but were not ESBL producers, based on Vitek 2 testing. Further testing showed that the majority were AmpC hyper-producing *Citrobacter* spp., the remainder being *E*. *coli* or *K*. *pneumoniae* that appeared to lack ESBL or AmpC activity and for which the basis of growth on the media was not known (Table 2). The *Enterobacter cloacae* did not contain a gene encoding an ESBL based on in silico PCR of whole genome sequence data for this isolate (data not shown).

In summary, plating after pre-enrichment followed by 24-hour incubation combines high sensitivity with a 48-hour turnaround time, and using this method, both ESBL *Brilliance* and chromID agars had comparable sensitivity and selectivity for the isolation of ESBL-producing Enterobacteriaceae from stool. There was a loss of selectivity after 48-hour incubation for all methods and when using pre-enrichment due to increased growth of non–ESBL-producing organisms.

The following is the supplementary data related to this article.

Supplementary Table 1

Supplementary data to this article can be found online at http://dx.doi.org/10.1016/j.diagmicrobio.2015.11.008.

## Figures and Tables

**Table 1:** Sensitivity and selectivity of two chromogenic agars for the isolation of ESBL-producing Enterobacteriaceae from 298 stool samples.

	ChromID ESBL direct plating		*Brilliance* ESBL direct plating		ChromID ESBL plating after enrichment		*Brilliance* ESBL plating after enrichment	
Incubation time (h)	24	48	24	48	24	48	24	48
No. of plates growing ESBL-producing organisms	73	87	68	79	112	113	114	115
Sensitivity^a^	63%	75%	59%	68%	97%	97%	98%	99%
No. of plates NOT growing organisms other than ESBL producers	252	210	258	192	189	163	182	172
Selectivity^b^	85%	70%	87%	64%	63%	55%	61%	58%

a Sensitivity was calculated by comparing the number of positive plates for a given method, compared with the cumulative yield of ESBL-producing organisms from all 4 methods (direct plating onto 2 media and plating onto both following a pre-enrichment step, n = 116/298).

b Selectivity for each method was defined as the number of plates that suppressed the growth of organisms that were not ESBL producers.

**Table 2:** Details of non–ESBL-producing organisms growing on 2 chromogenic agars.^a^

	ChromID ESBL direct plating		*Brilliance* ESBL direct plating		ChromID ESBL plating after enrichment		*Brilliance* ESBL plating after enrichment	
Incubation time (h)	24	48	24	48	24	48	24	48
*Pseudomonas* spp.	17	52	28	83	65	86	91	102
Other Gram-negative nonfermenters	4	6	1	3	13	15	2	2
Enterobacteriaceae								
AmpC^+^*Citrobacter* spp.	12	15	7	13	21	22	21	21
*E*. *coli*	1	1	0	1	2	3	2	2
*K*. *pneumoniae*	0	0	0	0	0	1	1	1
*E*. *cloacae*	0	0	0	0	1	1	1	1
Gram-positive cocci	28	34	4	8	30	34	1	3
Yeasts	0	0	0	0	0	1	0	0

a Some plates grew more than 1 species, and the total may not tally with sensitivity results shown in Table 1, which used agar plate as the denominator.
